# Seronegative Hepatitis C Virus Infection

**DOI:** 10.1007/s00005-013-0257-7

**Published:** 2013-11-09

**Authors:** Justyna Kaźmierczak, Agnieszka Pawełczyk, Kamila Caraballo Cortes, Marek Radkowski

**Affiliations:** Department of Immunopathology of Infectious and Parasitic Diseases, Medical University of Warsaw, Warsaw, Poland

**Keywords:** HCV, Anti-HCV, HCV RNA, Seronegative infection, Diagnostics

## Abstract

Hepatitis C virus (HCV) is a major cause of liver disease worldwide. The routine diagnostics identifying HCV infection include testing for specific anti-HCV antibodies by enzyme-linked immnunosorbent assay and viral genetic material in serum or plasma. However, a small proportion of patients persistently infected with HCV, in whom anti-HCV are undetectable, constitute a serious diagnostic and possibly epidemiologic problem, as they could facilitate pathogen spread in the population. This type of infection is termed seronegative or serosilent. Seronegative HCV infection is currently of great interest to both scientists and physicians. The review presents epidemiological data concerning the prevalence of seronegative HCV infection in HIV/HCV co-infected individuals, hemodialysis patients, and blood and organ donors. The possible mechanisms behind this atypical course of infection are discussed. Furthermore, the differences between seronegative and occult infections and prolonged seroconversion are explained.

## Introduction

Hepatitis C virus (HCV) is an etiologic factor of acute and chronic hepatitis, liver cirrhosis and hepatocellular carcinoma (Flisiak et al. 2011; Hu and Tong 1999). The estimated number of humans infected with the virus reaches 170 million worldwide including 0.7 million in Poland (Flisiak et al. 2011; Zagożdżon et al. 2009). Although HCV is a primary hepatotropic pathogen, its replication has been demonstrated in other cells, most notably in peripheral blood mononuclear cells (PBMC) and macrophages (Azzari et al. 2008; Chary et al. 2012; Laskus et al. 2000, 2004; Page et al. 2010; Revie and Salahuddin 2011; Zayed et al. 2010).

Diagnostic criteria for hepatitis C virus infection include the presence of anti-HCV antibodies and viral genetic material (HCV RNA) in blood serum. However, in some patients, the anti-HCV antibodies are absent despite the persistent presence of HCV RNA. Such status is termed “seronegative” or “serosilent” HCV infection and may be associated with clinical conditions such as human immunodeficiency virus (HIV) co-infection, hemodialysis and organ transplantation, but sporadically it is also seen in blood donors and other patients (Brojer et al. 2001; Schneeberger et al. 1998; Thomson et al. 2009; Tugwell et al. 2005).

Seronegative HCV infection represents a serious clinical and epidemiological problem and the pathogenetic mechanisms underlying this condition are poorly understood.

### Hepatitis C Virus Diagnostics

Routinely used diagnostic methods for HCV infection are based on the detection of anti-HCV antibodies and HCV RNA in blood serum (Pawlotsky et al. 1998). At present, antibodies are detected by third-generation enzyme immunoassay (EIA) or enzyme-linked immunosorbent assay (ELISA) using recombinant core and non-structural proteins NS3, NS4 and NS5 HCV (Albeldawi et al. 2010; Mondelli et al. 2005; Muñoz Espinosa 2002). To confirm positive serological test result, recombinant immunoblot assay (RIBA) test was routinely used (Pawlotsky et al. 1998; Van der Poel et al. 1991). However, molecular tests verifying the presence of HCV RNA are currently recommended (Tuaillon et al. 2010). The main limitation of diagnostic tests, based on the detection of antibodies (ELISA, RIBA), is lack of HCV infection identification during the serological window phase, i.e., during the period when the antibody level is below the detection limit of these tests (Nübling et al. 2002). This period ranges from 6 weeks (Morand et al. 2001; Thomson 2009) to 9 months (Arrojo et al. 2003), but is usually no longer than 12 weeks (Lauer and Walker 2001; Schreiber et al. 1996). It should be noted that some individuals with immunosuppression need even more time to seroconvert. Currently used tests with improved sensitivity have narrowed the serological window period following a primary acute-phase infection. The challenge is how to interpret partial or incomplete antibody seroconversions characterized by indeterminate test results on supplemental immunoblot assays (Carreño et al. 2012).

In contrast, HCV genetic material may be detected by means of molecular methods (NAT-nucleic acid testing) as early as 7–10 days post-infection (Schröter et al. 1997; Takedai et al. 2008). They include qualitative polymerase chain reaction (PCR), transcription-mediated amplification (TMA) and quantitative bDNA assay (branched-chain amplification) and real-time PCR (Scott and Gretch 2007).

Molecular methods are characterized by high specificity and sensitivity (detection limit up to 5 IU/mL); (Ross et al. 2001; Scott and Gretch 2007). In contrast to serological methods, molecular tests enable detection of viral infection during the very early phase (serological window period). They are used to confirm HCV infection, measure viral load and determine viral genotype necessary for treatment decisions. Finally, they allow monitoring the effectiveness of antiviral treatment (Higuchi et al. 2002; Stapleton et al. 1999).

### Pathogenesis of Seronegative HCV Infection

In most cases, both anti-HCV antibodies and HCV RNA are detectable in serum (Lok and Gunaratnam 1997; Pondé 2013). However, in some subjects anti-HCV antibodies are not detectable, even during the late infection phase. Reasons for this unusual condition may be delayed seroconversion (longer than 3 months post-infection), which sometimes may be prolonged to several years (Stapleton et al. 1999). The latter phenomenon is frequently found in patients with immune dysfunctions (immunosuppression) and intravenous drug users (Arrojo et al. 2003; Beld et al. 1999). In the latter group, anti-HCV antibodies may become detectable after superinfection with a different HCV genotype strain (Beld et al. 1999).

It is likely that prolonged or delayed seroconversion period may be due to low HCV viral load levels not sufficient for effective stimulation of the immune system. Low HCV antigen levels were reported to be a cause of weak recognition of the viral antigens by T helper cells and, consequently, low or absent B cell response (Mientjes et al. 1991).

The seronegative HCV infection due to the lack of production of specific anti-HCV antibodies is different from the situation in which the antibodies are synthesized and present in the blood, but in amounts not detectable by routinely used diagnostic tests. For instance, the sensitivity of third-generation EIA test is 97–99.9 %, which is very high but not complete (Ellethy et al. 2012).

Another reason for seronegativity may be the presence of immune complexes of antibodies and viral antigens, resulting in very low levels of free, circulating antibodies. This may occur in patients with relatively low anti-HCV response with simultaneous massive virus replication and antigen production. To overcome this problem, procedures for dissociation of immune complexes to “liberate” antibodies are available, although they are not routinely employed (Alnaqy et al. 2006; Pawlotsky et al. 1996; Troisi and Hollinger 1997).

While the majority of studies concerning seronegative HCV infection are focused on patients displaying immune dysfunctions, such as hypogammaglobulinemia (George et al. 2002; Post et al. 2004; Rossi et al. 1997), subjects co-infected with HIV or transplant organ recipients (Bonacini et al. 2001; Larouche et al. 2012; Wright et al. 1994), some authors suggested that inherent properties of particular HCV strains may be responsible. Thus, seronegative HCV infection may be a result of the presence of viral antigenic variants which stimulate the production of antibodies not recognized in standard serologic tests (Alnaqy et al. 2006; Pawlotsky et al. 1996). Moreover, some HCV strains may evade host immune responses or directly infect cells of the immune system (T and B cells); (Larouche et al. 2012; Sarhan et al. 2012). Genetic predispositions or defects affecting humoral responses against HCV were also suggested (Alnaqy et al. 2006; Larouche et al. 2012; Pawlotsky et al. 1996). Finally, it was also documented that seronegative infection may develop as a result of immune tolerance to HCV antigens, acquired in early life through vertical HCV transmission (Larouche et al. 2012).

### Epidemiology of Seronegative HCV Infection

The majority of epidemiological studies of seronegative HCV infection refer to HIV co-infected individuals, hemodialyzed patients, and organ and blood donors. The prevalence of this phenomenon is summarized in Table 1.

**Table 1:** The prevalence of seronegative HCV infection

Population	Seronegative HCV infection (%)	References
HIV/HCV co-infected individuals	3.2–13.2	Bonacini et al. (2001), Chamie et al. (2007), George et al. (2002), Hadlich et al. (2007)
Hemodialyzed patients	1–15	Carneiro et al. (2001), Fabrizi et al. (1999), Hanuka et al. (2002), Schröter et al. (1997)
Organ donors	0.2–0.9	Aswad et al. 2005, Challine et al. 2004, Legler et al. 2000
Blood donors	0.0004–0.08	Brojer 2005, Seyfried et al. 2005, Stramer et al. 2004

#### HIV/HCV Co-Infection

The concomitant infection with HIV and HCV is common, particularly among intravenous drug users and hemophiliacs, and ranges at 7–85 % (George et al. 2002; Vachon et al. 2008). For this reason, all HIV-infected patients should be monitored for the presence of anti-HCV antibodies (Hadlich et al. 2007; Vachon et al. 2008).

Some HCV RNA-positive HIV co-infected patients remain anti-HCV negative long after exposure. The lack of anti-HCV seroconversion in these patients is believed to be due to immune dysfunction secondary to HIV infection (Chamie et al. 2007; Sorbi et al. 1996).

In a pooled analysis of four studies, the frequency of HCV RNA positivity and concomitant absence of anti-HCV antibodies among HIV-infected patients was 3.2 % (Chamie et al. 2007); in one study, it was reported to be as high as 13.2 % (Bonacini et al. 2001; George et al. 2002; Hadlich et al. 2007). Seronegative HCV infection in HIV-infected individuals is more common in patients with CD4^+^ cells count below 100/μL and more advanced immunodeficiency (Hadlich et al. 2007). Among intravenous drug users exhibiting increased levels of alanine aminotransferase activity and decrease of CD4^+^ cells counts below 200/μL, the prevalence of seronegative infection is as high as 24 % (Chamie et al. 2007). This indicates that molecular techniques should be widely employed as first-line diagnostic methods, especially among patients with elevated activity of liver enzymes (George et al. 2002).

#### Hemodialyzed Patients

Hemodialyzed patients are a high-risk group for parenterally transmitted infections, including HCV infection. Immune dysfunctions are common among these patients and include aberrant B cell activation resulting in delayed synthesis of antibodies and/or their low levels in blood (Fabrizi et al. 2012; Schneeberger et al. 2000). It is estimated that 1–60 % of hemodialyzed patients are HCV infected (Hanuka et al. 2002; Kelley et al. 2002). The presence of HCV RNA among seronegative patients is found in 1–15 % of the cases (Carneiro et al. 2001; Fabrizi et al. 1999; Hanuka et al. 2002; Schröter et al. 1997).

The high prevalence of seronegative HCV infections in this group of patients warrants wider use of molecular tests for diagnostic purposes.

#### Organ and Blood Donors

Despite many precautions and procedures reducing the transmission of infections, organ transplantation still carries a risk of HCV infection. Among anti-HCV negative organ donors, HCV RNA is detectable in less than 1 % (0.2–0.9 %); (Aswad et al. 2005; Challine et al. 2004); however, immune dysfunction present in transplant recipients entails an increased risk of infection. Molecular testing for HCV RNA and viral core antigen are not routinely performed in organ donor candidates, mostly because of high costs and complex diagnostic procedures (Challine et al. 2004). However, since a single donor can be a source of graft material for multiple recipients, the prospective donors should be carefully screened for HCV infection with molecular methods (Tugwell et al. 2005).

According to different sources, the frequency of seronegative HCV infection among blood donors in developed countries ranges at 0.0004–0.08 % (Brojer 2005; Legler et al. 2000; Seyfried et al. 2005; Stramer et al. 2004). One of the most important means to control the spread of HCV infection is molecular testing of donors’ blood (Brojer 2005; Letowska et al. 2009). Detection of anti-HCV antibodies in blood donors has been introduced in the early 1990s of the last century (Seyfried et al. 2005).

Molecular testing, aimed at the detection of HCV infection, was widely criticized for its high cost. However, since the year 2000 it is obligatory in the majority of countries, including Poland (Alnaqy et al. 2006; Brojer 2005; Challine et al. 2004). Its introduction for each blood collection significantly increased the safety of blood and blood-derived product transfusion (Alnaqy et al. 2006; Brojer 2005). For economic reasons, pooled testing of plasma collected from donors (48 or more) has been routinely performed. In case HCV RNA is detected in the pooled specimens, the individual samples are tested separately. Currently used procedures recommend performing molecular testing of single samples; however, there are variations depending on the laboratory and kits employed. It should be stressed that “dilution” of material when mixing multiple samples may lead to false-negative results especially when single HCV-positive sample with low viremia is present in the pool (Brojer 2005). One can expect that simplification and cost reduction of molecular tests will promote their universal use.

### Specific Memory Cells in HCV Infection

Potent CD4^+^ and CD8^+^ cellular responses specific to HCV antigens may lead to virus elimination (Bertoletti and Ferrari 2003; Chang 2003; Meyer et al. 2007; Thimme et al. 2001; Zhang et al. 2009). It was demonstrated that individuals eliminating HCV infection display stronger T cell responses than those chronically infected (Lauer and Walker 2001; Lechner et al. 2000; Meyer et al. 2007; Urbani et al. 2006). Strong and long-lasting CD4^+^ and CD8^+^ responses are present in more than 80 % of patients who cleared HCV infection (Zhang et al. 2009). T cell responses may persist as long as 20 years after viral clearance providing evidence of past HCV infection (Takaki et al. 2000; Wedemeyer et al. 2002).

Specific cellular responses were also observed among high-risk groups for HCV infection and/or subjects exposed to HCV who remained persistently negative for HCV RNA and anti-HCV antibodies. Examples include sexual partners and family members of HCV-infected patients and laboratory workers (Bronowicki et al. 1997; Koziel et al. 1997; Scognamiglio et al. 1999). These cases may represent specific immune memory after a subclinical course of infection and subsequent spontaneous viral clearance (Scognamiglio et al. 1999).

The absence of specific anti-HCV antibodies and HCV RNA in subjects displaying specific cellular responses may be a result of exposure dose that was insufficient to induce viral hepatitis or even trigger humoral response. Broad cellular responses to many HCV antigens were also found in seronegative prisoners who were transiently HCV RNA positive in blood, but eventually cleared the infection. It was also suggested that the absence of anti-HCV antibodies could be a consequence of massive cytotoxic T responses directed against virus-infected B cells (Scognamiglio et al. 1999).

According to some studies, HCV elimination is not necessarily the result of strong cellular responses, as some patients who spontaneously cleared the virus showed weak or even absent CD4^+^ and CD8^+^ responses against viral antigens (Meyer et al. 2007).

### Occult HCV Infection

“Occult HCV infection” is characterized by the absence of anti-HCV antibodies and HCV RNA in the blood with concomitant presence of viral genetic material in hepatocytes and elevated activity of liver enzymes indicating an ongoing inflammatory process in the liver (Castillo et al. 2004; Comar et al. 2006; Esaki et al. 2004; Quiroga et al. 2006, 2009). The term “occult HCV infection” is also sometimes used in case of individuals who cleared HCV infection, but display the presence of HCV RNA in liver tissue and anti-HCV antibodies in serum (Carreño 2006). The latter should perhaps be considered a particular clinical representation of chronic HCV infection characterized by low replication, or a late stage of infection directly preceding viral elimination.

Typical features of occult HCV infection include lower liver enzymes activity and lower percentage of infected hepatocytes than is typical for clinically overt infection (Pardo et al. 2007). Most patients with occult HCV infection display specific CD4^+^ and CD8^+^ responses and, surprisingly, their memory T cell activation is much more frequent than in those with typical clinical course. It is suspected that these cells may be involved in viral replication control to undetectable levels (Quiroga et al. 2006).

The estimated frequency of occult HCV infection among patients displaying hepatic dysfunction may be as high as 57 % (Castillo et al. 2004). In the majority of them HCV RNA is also present in PBMC (70 %), and in over half of the latter (61 %) markers of active viral replication were detected in this extrahepatic compartment (Castillo et al. 2004, 2005).

Due to the difficulties in detecting occult HCV infection with standard laboratory tests, all data regarding this phenomenon come from research studies. Nevertheless, this form of infection seems to be distributed worldwide and its prevalence among the healthy population was reported to be as high as 3.3 % (Carreño et al. 2012; De Marco et al. 2009) and even higher among patients suffering from cryptogenic hepatitis or cryptogenic cirrhosis with hepatocellular carcinoma (10–74 %); (Bokharaei-Salim et al. 2011; Comar et al. 2006; Esaki et al. 2004; Idrees et al. 2011; Zaghloul and El-Sherbiny 2010).

## Conclusions

Seronegative HCV infection represents a significant clinical problem. One possible consequence of seronegative infection may be a continuous transmission of HCV in the low-risk population (Larouche et al. 2012; Stapleton et al. 1999; Wiese et al. 2000).

The pathogenesis of seronegative HCV infection is not fully understood. Most likely it is a result of immune dysfunctions resulting in the inability to properly recognize HCV antigens and produce specific antibodies. Another mechanism could be infection with atypical viral strain (Alnaqy et al. 2006; Beld et al. 1999; Pawlotsky et al. 1996).

To optimize HCV diagnostics, including seronegative infections, the following issues must be considered: patient’s immune status, type of biological sample collected, time point of sample collection, possibility of delay in antibody production and the sensitivity limit of different diagnostic tests. The diagnostic procedures based on high sensitivity molecular HCV RNA detection should be more widely introduced, especially among high risk groups and organ donors.

As viral blood levels typically usually fluctuate and may temporarily drop below the level of detection, in some initially HCV RNA-negative cases the testing should be repeated. In patients with persistent hepatic dysfunction of unknown etiology, the search for viral RNA in samples other than blood (most notably liver and PBMC) should be considered. Although cumbersome in clinical practice, these procedures could contribute to improvements in HCV infection detection and consequently facilitate the development of strategies aimed at decreasing the transmission frequency of this virus.

## Abbreviations

EIA: Enzyme immunoassay
ELISA: Enzyme-linked immunosorbent assay
HCV: Hepatitis C virus
HCV RNA: Hepatitis C viral genetic material
HIV: Human immunodeficiency virus
PBMC: Peripheral blood mononuclear cells
RIBA: Recombinant immunoblot assay
PCR: Polymerase chain reaction
